# Space Maintainers Used in Pediatric Dentistry: An Insight of Their Biosecurity Profile by Applying In Vitro Methods

**DOI:** 10.3390/ma14206215

**Published:** 2021-10-19

**Authors:** Magda Mihaela Luca, Malina Popa, Claudia G. Watz, Iulia Pinzaru, George Andrei Draghici, Ciprian V. Mihali, Cristina Adriana Dehelean, Roxana Buzatu, Camelia Szuhanek

**Affiliations:** 1 Department of Pedodontics, Faculty of Dental Medicine, “Victor Babeş” University of Medicine and Pharmacy Timisoara, 9 No., Revolutiei Bv., 300041 Timişoara, Romania; luca.magda@umft.ro (M.M.L.); popa.malina@umft.ro (M.P.); 2 Pediatric Dentistry Research Center, Faculty of Dental Medicine, “Victor Babeş” University of Medicine and Pharmacy Timisoara, 9 No., Revolutiei Bv., 300041 Timişoara, Romania; 3 Departament of Pharmaceutical Physics and Biophysics, Faculty of Pharmacy, “Victor Babeş” University of Medicine and Pharmacy Timisoara, 2nd Eftimie Murgu Sq., 300041 Timişoara, Romania; farcas.claudia@umft.ro; 4 Research Center for Pharmaco-Toxicological Evaluations, Faculty of Pharmacy, “Victor Babes” University of Medicine and Pharmacy Timisoara, Eftimie Murgu Square No. 2, 300041 Timişoara, Romania; cadehelean@umft.ro; 5 Departament of Toxicology and Drug Industry, Faculty of Pharmacy, “Victor Babeş” University of Medicine and Pharmacy Timisoara, 2nd Eftimie Murgu Sq., 300041 Timişoara, Romania; 6 Department of Life Sciences, Faculty of Medicine, “Vasile Goldis” Western University of Arad, 86 No., Liviu Rebreanu St., 310414 Arad, Romania; mihaliciprian@yahoo.com; 7 Molecular Research Department, Research and Development Station for Bovine, 32 No., Bodrogului St., 310059 Arad, Romania; 8 Department of Dental Aesthetics, Faculty of Dental Medicine, “Victor Babeş” University of Medicine and Pharmacy Timisoara, 9 No., Revolutiei Bv., 300041 Timişoara, Romania; drbuzaturoxana@gmail.com; 9 Department of Orthodontics, Faculty of Dental Medicine, “Victor Babes” University of Medicine and Pharmacy Timisoara, 300041 Timişoara, Romania; cameliaszuhanek@umft.ro; 10 Orthodontic Research Center, Faculty of Dental Medicine, “Victor Babeş” University of Medicine and Pharmacy Timisoara, 9 No., Revolutiei Bv., 300041 Timişoara, Romania

**Keywords:** metallic biomaterials, keratinocytes, antimicrobial activity, morphology, cytotoxicity, gene expression

## Abstract

Space maintainers have presented an increased interest due to their chemical composition which influences the electrochemical and electrolytic processes of the oral cavity, leading to important biological activity. The present study was purported to evaluate the biological in vitro activity of three types of space maintainers (S1, S2, and S3, differing from each other in terms of metal composition) used in pediatric dentistry, in terms of their antimicrobial effect and biosecurity profile using two types of keratinocytes (PGK: primary gingival keratinocytes, and HaCaT: human immortalized keratinocytes) by assessing the morphology, viability, cytotoxicity, and gene expression of the cells. Statistical differences were calculated by the one-way ANOVA test, followed by Tukey’s post-test. Antimicrobial screening highlighted a dilution-dependent influence that, in the case of all strains tested, did not show inhibition or stimulation of bacterial growth. The in vitro evaluations revealed that the test samples did not induce important cytotoxic potential on both keratinocyte cell lines (HaCaT and PGK), with the cells manifesting no morphological alteration, a good viability rate (above 90%: PGK–S1, * *p* < 0.05), and a low cytotoxic activity (less than 11%: PGK, S1 *** *p* < 0.001 and S3 * *p* < 0.05; HaCaT, S1 ** *p* < 0.01). The data obtained in this study highlight the fact that the samples analyzed are biocompatible and do not develop the growth of the studied bacteria or encode the gene expression of primary and immortalized keratinocytes.

## 1. Introduction

Metallic materials, especially those made of stainless steel, are very commonly used in dental practice for both children and adults. Space maintainers are mainly employed in pediatric dentistry for preserving the spaces left after the extraction of the first two molars, in order to prevent a set of subsequent complications such as: atopic eruption; agglomeration; malocclusion; etc. [1,2]. The use of these space maintainers, either fixed or detachable, has not only advantages, but also some disadvantages, such as allergies or possible plaque accumulation, a process that underlies the formation of dental caries, and even the development of periodontal disease in a case of poor oral hygiene [3,4]. Dental plaque is maintained by a biofilm which provides, through a primitive system of vessels, nutrients to bacteria, and is the main cause of gingival inflammation and periodontitis. Orthodontic metal objects can maintain dental plaque, and reversible, or even irreversible, processes can take place during orthodontic treatments [1,5]. The use of space maintainers contributes to changes in the oral cavity that are determined by the influence of metallic materials on pH, oral environment, buffer capacity, and others. It was also stated that there is a possibility of developing bacterial concentration, especially streptococci, associated with early demineralization, and lactobacilli, associated with the development of lesions [6]. Studies in the literature are very varied: some of them support the maintenance of bacterial cultures, and others are on the contrary, and have not identified changes in bacterial cultures. *Enterococcus faecalis*, an anaerobic gram-positive coccus, is the main bacterium that leads to the failure of endodontic treatments, and is also associated with periodontal disease. It has also been recognized for its resistance to antimicrobial treatments [7]. Increased interest has also been given to the involvement of *Candida albicans* in orthodontic treatments, a fungus with a possible role in the etiology of caries in healthy patients [8]. In the oral cavity, electrochemical and electrolytic processes are intensified due to the aerobic environment, heat, and moisture, which can lead to biodegradation of metals. Possible concerns are raised especially by the release of metallic ions and different particles in case of biodegradation [9]. Nickel can produce both local and systemic toxicity, manifested by allergenic, immunogenic, chemostatic, and even mutagenic effects [10,11].

Space maintainers are of real use in pediatric dental practice, but long-term use is a challenge because they can lead to the development of certain oral diseases that are based on different microbes [12]. The use of these types of metal materials in young patients can raise a number of problems due to: (i) the development of bacterial plaque; or (ii) the appearance of more serious problems based on dental hygiene, which are difficult to control. Different studies on orthodontic appliances have been conducted [13,14,15], but data targeting space maintainers, especially those based on biological activity, are limited. Biologically testing them is useful in order to be able to contribute them to the techniques of prolonging the life span.

Regarding this aspect, the present study was purported to evaluate biological in vitro activity of three type of space maintainers, as follows: (i) antimicrobial activity by agar diffusion method; and (ii) in vitro biocompatibility by using two different keratinocyte cell lines, and performing basic in vitro assays (cell morphology assessment, cell viability evaluation, and cytotoxic effects) together with possible changes in gene expression of keratinocytes when the cell cultures are exposed to a dilution of 1:15 (*v*:*v*%) extraction saliva:cell culture medium for an interval of 24 h.

The in vitro model used in the present study is represented by two different cell lines: human primary gingival keratinocytes (PGK); and human immortalized keratinocytes (HaCaT). The selection of these two cell lines is based on the fact that primary cells develop closer behavior to the in vivo keratinocytes when compared to the immortalized cell line, which may suffer several genotypic and phenotypic transformations to become more resistant in order to be suitable for long periods of cell culturing [16]. Thus, by performing the in vitro evaluations on the both types of keratinocytes (primary and immortalized), a more complex biological profile could be obtained showing comparative data between these two cell lines (PGK vs. HaCaT).

The in vitro methods employed in the current study have already proved to be relevant for offering preliminary data in establishing the biological profile of different dental materials [17,18,19], whereas keratinocyte cultures proved to be good candidates as in vitro models for providing the preliminary biosecurity of different products related to oral use [20,21,22].

## 2. Materials and Methods

**Figure 1:**
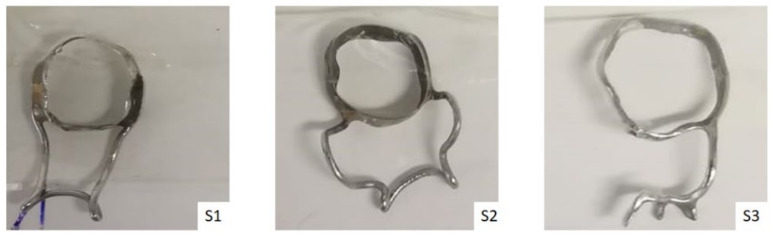
General aspects of space maintainers utilized in the study.

The present study focuses on the evaluation of dental metallic specimens used in pediatric dentistry. For this purpose, three types of band and loop space maintainers were fabricated, as can be seen in Figure 1. An S1-one band from 3 M Unitek (type: Victory series, narrow contoured, 902; alloy: stainless steel; chemical composition % by mass: 18–20% Cr, 8–12% Ni, 2.0% Mn (max), 1.0% Si (max), balance: Fe; loop made from 0.036-in. steel wire, and flux (Dentaurum Universal Dentaflux)) was used for loop soldering (Dentaurum Hartlot Hard solder). An S2-one band from Dentaurum (type: Dentaform DIN 1.4303 (Cr, Ni austenitic stainless steel); chemical composition % by mass: 17–19% Cr, 11–13% Ni, 2.0% Mn (max), 1.0% Si (max), balance: Fe; loop made from 0.036-in. steel wire, and flux (Dentaurum Universal Dentaflux)) was used for loop soldering (Dentaurum Hartlot Hard solder). An S3-one band and a loop space maintainer were fabricated using 3D printing with the use of a digital scanning and designing. To verify the accuracy of the future space maintainer, a polymeric resin framework was produced, and the final space maintainer was obtained by a titanium-based powder metal material and selective laser sintering technology.

The test dental metallic specimens were maintained in artificial saliva for 72 h, at 37 °C and 180 revolutions per minute (RPM) to simulate the physiological conditions of the oral cavity. This technique offers the possibility to evaluate the in vitro cytotoxicity of medical devices by means of extraction, according to International Standard Organization [23].

In brief, the extraction artificial saliva, containing the residual ions/substances released by test samples, was further used to obtain a dilution of 1:15 (*v*:*v*%) artificial saliva:cell culture medium, and the mixture was used to stimulate both cell lines: primary gingival keratinocytes (PGK); and immortalized human keratinocytes (HaCaT). The mixture was also used to evaluate the antimicrobial activity and changes in gene expression of the keratinocyte cell lines.

### 2.1. SEM Analysis

The surface topography of the test samples was analyzed by means of scanning electron microscopy (SEM) using the FEI Quanta 250 microscope (Eindhoven, Holland). Pictures were taken at two different magnification orders (24× and 500×) to allow optimal surface topography analysis.

### 2.2. Antimicrobial Assay

Different microorganisms (presented in Table 1) were treated with three dilutions (1:5, 1:10, and 1:15) of saliva samples. All strains were acquired from ATCC (American Type Culture Collection, Manassas, VA, USA). The agar diffusion assay for susceptibility testing was conducted in accordance with the Clinical & Laboratory Standards Institute (CLSI) standard [24].

Antimicrobial in vitro analysis was realized on 6 mm sterile paper disks which were treated with 20 μL of S0, S1, S2, and S3 (dilutions of 1:5, 1:10, and 1:15, 25). The method was conducted in accordance to the one previously described in the literature [25,26]. In brief, the following procedures were performed: a) preparation of standard dilutions, which consisted of 10^−3^ fresh bacteria and 10^−2^ fresh fungi cultures; b) cultivation and treatment on Petri plates with the S0, S1, S2, and S3; and c) incubation at optimal temperature (37 °C for bacteria and 30 °C for fungi) for 24–48 h. For positive controls represented by antibiotics, Gentamicin and Nystatin OxoidTM commercial disks, acquired from ThermoFischer Scientific (Waltham, MA, USA) were used, and distilled water was chosen as negative control. The experiments were realized in triplicate, and the presence of any zone of inhibition was noted.

### 2.3. Cell Lines and Cell Culture Conditions

Primary gingival keratinocytes (the PGK cell line) and human immortalized keratinocytes (HaCaT) cells were used in the present study to evaluate the biological profile of the test metallic samples. The PGK cell line was acquired from the American Type Culture Collection Manassas, VA, USA (ATCC, code no PCS200014™), together with all the reagents necessary for cell culturing, such as: Dermal Cell Basal Medium (ATCC^®^ PCS-200-030™); and Keratinocyte Growth Kit (ATCC^®^ PCS-200-040™). The HaCaT cell line was cultured in DMEM high glucose (4.5 g/L) media, with 15 mM HEPES, and 2 mM L-glutamine (Sigma-Aldrich, Munich, Germany), supplemented with 10% Fetal Calf Serum and 1% antibiotic mixture of 100 U/mL penicillin:100 µg/mL streptomycin. The in vitro procedures were performed under standard conditions: the cells were maintained in humidified atmosphere containing 5% CO_2_ and 37 °C, in a Steri-Cycle i160 incubator (Thermo Fisher Scientific Inc., Waltham, MA, USA). The in vitro experiments performed in the current study respected all the conditions required for cell cultures, such as: (i) cell growth under a humidified atmosphere enriched in 5% CO_2_ and 37 °C using the Steri-Cycle i160 incubator (Thermo Fisher Scientific, Inc., Waltham, MA, USA); and (ii) experiments being performed under sterile conditions using the MSC Advantage 12 model biosafety cabinet (Thermo Fisher Scientific, Inc., Waltham, MA, USA).

### 2.4. Cell Morphology Assessment

After exposure to the 1:15 (*v*:*v*%) extraction saliva:cell culture medium mixture, the morphological aspects of the PGK and HaCaT cell lines were evaluated by taking pictures at a magnification of 20×, initially (0 h) and after 24 h, using the Olympus IX73 inverted microscope equipped with DP74 camera (Olympus, Tokyo, Japan).

### 2.5. Cell Viability Assay by Means of Alamar Blue Test

The effect induced by the test samples on the primary gingival keratinocytes (PGK) was determined by applying the Alamar Blue colorimetric test, as previously described [27]. In brief, a density of 10^4^ cells/well were cultured onto 96-well plates, and incubated overnight. The next day, the old medium was removed, and the cells were stimulated with the 1:15 (*v*:*v*%) extraction saliva:cell culture medium mixture. The control cells were maintained in the same conditions as the stimulated ones, however, they were treated with a specific culture medium. To quantify the cell viability percentage, the absorbance of each well was determined spectrophotometrically at two wavelengths (570 and 600 nm) by means of a microplate reader (xMarkTM Microplate, Bio-Rad Laboratories, Hercules, CA, USA), and the following formula was applied [17,28]:

$$\mathrm{Cell}\;\mathrm{viability}\;\left(\%\right)=\frac{(\varepsilon_{\mathrm{OX}})\lambda_{2}\times A_{\mathrm{test}}{\;\lambda }_{1}-\left(\varepsilon_{\mathrm{OX}}\right)\lambda_{1}\times A_{\mathrm{test}}{\;\lambda }_{2}}{\left(\varepsilon_{\mathrm{OX}}\right)\lambda_{2}\times A_{0}\lambda_{1}-\left(\varepsilon_{\mathrm{OX}}\right)\lambda_{1}\times A_{0}\lambda_{2}}\times 100$$

where:

ε_ox_ = molar extinction coefficient of the oxidized Alamar blue reagent

A_test_ = absorbance of test wells

A_0_ = absorbance of control well

λ_1_ = 570 nm

λ_2_ = 600 nm

### 2.6. Lactate Dehydrogenase Assay

An LDH assay was employed to evaluate the cytotoxic effect induced by the extraction saliva of test samples (S1, S2, S3) on primary gingival keratinocytes (the PGK cell line) and immortalized human keratinocytes (the HaCaT cell line). The principle of this technique is based on quantification of the lactate dehydrogenase (LDH) into the extracellular medium. Since LDH is a cytosolic enzyme, the LDH leakage could be quantified only when the cellular membrane damage occurs, which is associated with a cytotoxic feature. The protocol performed for LDH analysis was similar to the one for the Alamar blue assay, described in Section 2.5, however some exceptions were present. On the day of the assay, 50µL/well of the stimulation medium was transferred into a new 96-well plate, and mixed with 50 µL/well of the reaction mixture. After that, the plate was incubated for 30 min at room temperature, and finally, the reaction was stopped by adding 50 µL/well of the stop solution. The LDH leakage into the extracellular medium was determined spectrophotometrically by reading the absorbances of each well at the wavelengths of 490 nm and 680 nm with a microplate reader (xMarkTM Microplate; Bio-Rad Laboratories, Inc., Hercules, CA, USA).

### 2.7. RT–PCR Protocol

To evaluate the impact of the dental metallic samples on gene expression, a RT–PCR was performed. The keratinocytes were grown on 6-well plates (10^6^ cells per well) overnight, and the next day, the cell monolayer was treated with the 1:15 (*v*:*v*%) extraction saliva:cell culture medium mixture for 24 h.

Total RNA was isolated using the Trizol reagent (Thermo Fisher Scientific, Inc., Waltham, MA, USA) and the Quick-RNA™ purification kit (Zymo Research, Irvine, CA, USA). Total RNA was further transcribed, and the experiment was conducted with Maxima^®^ First Strand cDNA Synthesis Kit (Fermentas, Thermo Fisher Scientific, Inc., Waltham, MA, USA). Quantitative real-time PCR analysis was realized by means of the Quant Studio 5 real-time PCR system (Thermo Fisher Scientific, Inc., Waltham, MA, USA) in the presence of Power SYBR-Green PCR Master Mix (Thermo Fisher Scientific, Inc., Waltham, MA, USA). The primer pairs (Thermo Fisher Scientific Inc., Waltham, MA, USA) used in this study are presented in Table 2.

### 2.8. Statistical Analysis

The GraphPad Prism 7 software (GraphPad Software, San Diego, CA, USA) was employed to perform and present the statistical analysis data. The one-way ANOVA test was applied to determine the statistical differences of test samples versus control, followed by Tukey’s post-test (* *p* <0.05, ** *p* < 0.01, *** *p* < 0.001, and **** *p* < 0.0001).

## 3. Results

### 3.1. SEM Evaluation of Space Maintainers’ Surface

**Figure 2:**
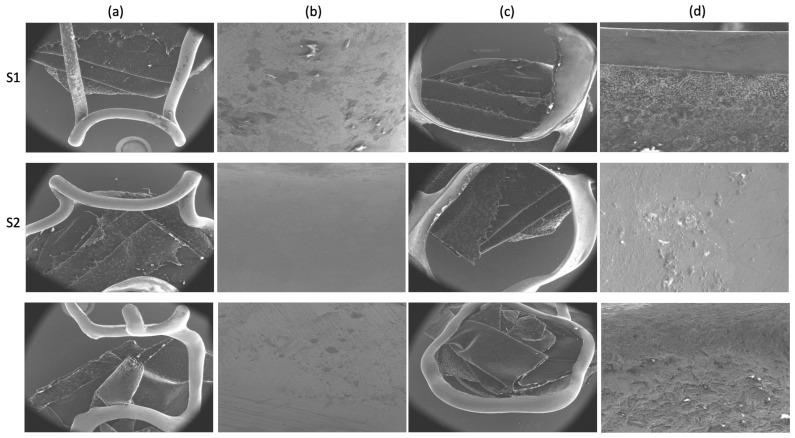
Surface morphology of S1 (3 M Unitek, type Victory series), S2 (Dentaurum; type Dentaform DIN 1.4303), and S3 (titanium-based powder metal material). (**a**) SEM image of the loop (24×); (**b**) SEM image of the loop (500×). (**c**) SEM image of the wire (24×); (**d**) SEM image of the wire (500×).

The qualitative surfaces topography of the test dental metallic samples is presented below in Figure 2: S1 (3 M Unitek, type Victory series); S2 (Dentaurum; type Dentaform DIN 1.4303); S3 (titanium-based powder metal material); loop surface details (a and b); and wire surface details (c and d). The changes in the surface of the samples are easily observed, the most uniform of these being the S3 sample.

### 3.2. Antimicrobial Evaluation

**Figure 3:**
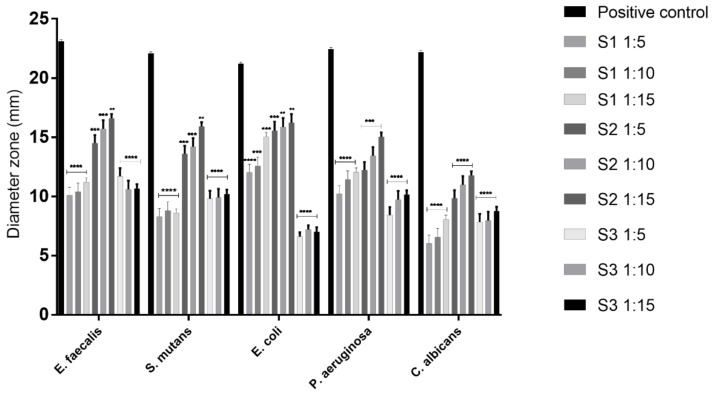
Antimicrobial evaluation of saliva samples from space maintainers at different dilutions in microorganisms by agar diffusion assay. The data are expressed as DZI (diameter zone of inhibition). The results are the mean values ± SD of three independent tests conducted in triplicate. One-way ANOVA analysis was used to establish the statistical differences in rapport with positive control followed by Tukey’s post-test; ** *p* < 0.01, *** *p* < 0.001, **** *p* < 0.0001.

The saliva samples were tested to observe the influence on the antimicrobial effect. The data revealed that the test products exerted low, or even a lack of, antimicrobial activity against all tested microorganisms in a concentration and type-dependent manner (Figure 3). In the case of *E. faecalis* and *S. mutans* (positive bacteria), the highest activity was observed for S2 at all three dilutions, whereas in the case of *E. coli* and *P. aeruginosa* (negative bacteria), S1 and S2 manifested almost similar activity. The antifungal action of S1, S2, and S3 exerted on *Candida albicans* was considerably lower compared to positive control.

### 3.3. Cell Morphology Assessment

**Figure 4:**
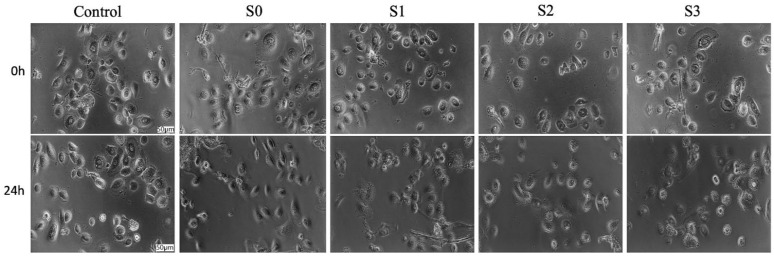
Morphological aspects of primary human gingival keratinocytes (PGK) at 0 h and 24 h post-stimulation with 1:15 (*v*:*v*%) extraction saliva:cell culture medium mixture. Pictures were taken at magnification of 20×; the scale bars represent 50 µm.

**Figure 5:**
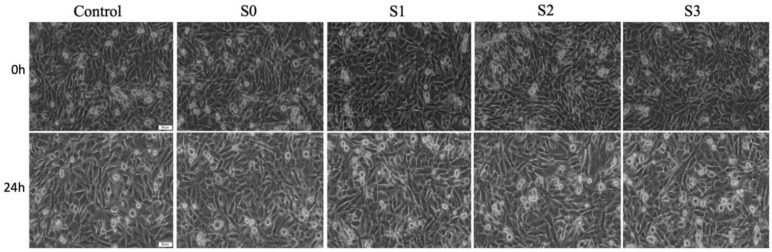
Morphological aspects of immortalized human keratinocytes (HaCaT) at 0 h and 24 h post-stimulation with 1:15 (*v*:*v*%) extraction saliva:cell culture medium mixture. Pictures were taken at magnification of 20×; the scale bars represent 50 µm.

The morphological aspects of both keratinocyte cell lines (PGK an HaCaT) were evaluated at 24 h post-stimulation, and the cell features observed under these conditions were compared to the morphological characteristics manifested by the treated cells at 0 h (initial time of exposure), as well as to the morphological aspects presented by the control cells (cells treated with the specific cell culture medium) (Figure 4 and Figure 5). PGK control cells present a rounded shape with a cobblestone-like appearance. Regarding the morphological aspects manifested by the PGK-treated cells, no significant alterations were recorded, as presented in Figure 4. Also, up to 80% confluence was noted, indicating these cells are growing in colonies, a feature that is respected by all the cells (control cells and cells exposed to test samples).

The HaCaT cell line is a highly adherent cell line, and the cells present an elongated shape. The control HaCaT cells present a high confluence (above 95%), and this confluence is maintained even after addition of test samples for a contact time of 24 h. Nevertheless, no important cytopathic features were manifested by the cells exposed to test samples for 24 h when compared to the cellular characteristics presented by the cells at the initial time of test samples application (0 h), or by the control cells, as depicted in Figure 5.

### 3.4. Cell Viability Assesment

**Figure 6:**
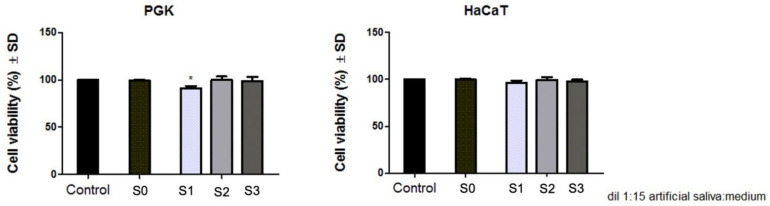
Primary human gingival keratinocytes (PGK) and immortalized human keratinocytes (HaCaT) viability after treatment with 1:15 (*v*:*v*%) artificial saliva:cell culture medium mixture for 24 h. Blank (S0) is represented by artificial saliva; S1, S2, and S3 represent the extraction saliva of test dental specimens. The results are presented as cell viability percentage (%) normalized to control cells. The graph bars are expressed as mean values ± SD of three independent experiments. One-way analysis of variance (ANOVA) test was performed to determine the statistical differences of test sample versus control, followed by Tukey’s multiple comparisons post-test (* *p* < 0.05).

To evaluate the effect induced by dental metallic specimens (S1, S2, S3) on primary gingival keratinocytes (PGK) and immortalized human keratinocytes (HaCaT), the Alamar blue proliferation test was performed at 24 h post-treatment, and the results obtained are presented in Figure 6.

The results indicate that both primary and immortalized keratinocytes manifested no significant cell viability reduction after exposure to the artificial saliva of the test dental samples (S1, S2, S3) when a dilution of 1:15 (*v*:*v*%) extraction saliva:cell culture medium was applied for an incubation time of 24 h. However, among the three test samples, S1 induced the most reduced cell viability rate, with the PGK cell line manifesting a viability of 91.07%, whereas the HaCaT cell line showed a viability percentage of 96.4%. Regarding the biosafety of the other two test dental specimens (S2 and S3), both keratinocyte cell lines manifested a viable cell population above 98%, under the same experimental conditions (1:15 (*v*:*v*%) dilution of extraction saliva in cell culture medium and an exposure time of 24 h).

### 3.5. Evaluation of the Cytotoxic Effect through LDH Release Method

**Figure 7:**
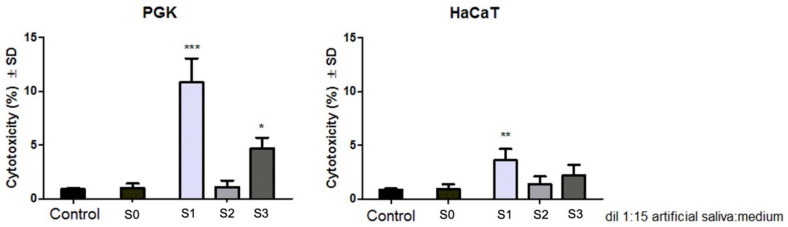
Cytotoxicity percentage of primary gingival keratinocytes (PGK) cells and immortalized human keratinocytes (HaCaT), at 24 h post-treatment with 1:15 (*v*:*v*%) artificial saliva:cell culture medium mixture. Blank (S0) is represented by artificial saliva; S1, S2, S3 represent the extraction saliva of test dental specimens. The results represent the mean values ± SD of three separate experiments. One-way ANOVA test was performed to determine the statistical differences followed by Tukey’s multiple comparisons analysis (* *p* < 0.05, ** *p* < 0.01 and *** *p* < 0.001).

The lactate dehydrogenase (LDH) assay revealed that the S1 sample induced the most important cytotoxic effect on both keratinocyte cell lines, revealing a cytotoxic rate of 10.84% on the PGK cell line, whereas the HaCaT cell line was more resistant to the test compounds treatment, exhibiting a cytotoxicity of 3.62% when S1 sample was tested for 24 h exposure time. The second most cytotoxic sample is represented by S3, which induced a cytotoxicity of 4.68% on primary keratinocytes and a cytotoxic rate of 2.20% on immortalized keratinocytes. The S2 test sample showed the lowest toxicity, inducing a cytotoxic rate below 1.5% on both keratinocyte cell lines, as presented in Figure 7.

### 3.6. RT–PCR

**Figure 8:**
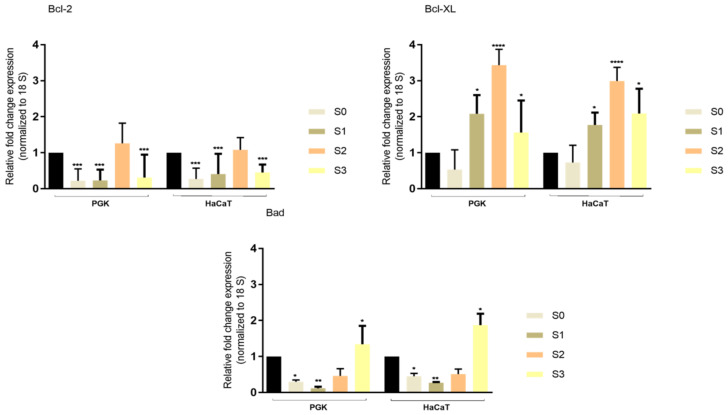
Relative fold expression of mRNA expression of pro-apoptotic Bad marker, and anti-apoptotic Bcl-2 and Bcl-XL markers in primary gingival keratinocytes (PGK) cells and immortalized human keratinocytes (HaCaT), after exposure to saliva test samples. The mRNA expression levels were normalized to 18 S expression. The mean values ± SD of three independent experiments are presented, and one-way ANOVA with Tukey’s post-test was used to evaluate the statistical differences (* *p* < 0.05, ** *p* < 0.01, *** *p* < 0.001, and **** *p* < 0.0001).

Figure 8 pointed out gene expression levels in primary gingival and immortalized keratinocytes in response to exposure to test saliva samples after 24 h incubation by RT–PCR analysis. The S1 and S3 mRNA expression levels were significantly lower than those on the S2 for anti-apoptotic marker Bcl-XL, whereas for the pro-apoptotic marker “Bad”, S3 presented the higher expression level, as can be seen in Figure 8.

## 4. Discussion

The biosafety profile of space maintainers is closely related to the row material from which these items are manufactured, as the release of ions (especially metal ions, such as Ti, Ni, V, Al, Cr, and Co) from dental metallic devices represents a debatable topic due to the hypothesized allergenic, cytotoxic, or mutagenic reactions [29,30]. The main factor responsible for the release of metal ions is associated with the corrosion phenomenon that can be induced by pH variation, oral temperature, and mechanical stress, however it can also be caused by oral microflora, and the so-called biocorrosion [30]. Even if the corrosion resistance of dental metal alloys (stainless steel, type 316 L; Ti-based alloy; CP Ti and Ti-6Al-4V) is usually very high [31], due to the various oral conditions to which dental metallic devices are exposed to, localized corrosion reactions may occur representing favorable aspects for metal ions release. As described by Fernandez-Minano et al., Ref. [32] low pH may have a high corrosive potential on Ni-based materials, and also, a diet rich in carbonated beverages and chlorides may facilitate the corrosion process by interacting with the chromium oxide layer of stainless steel devices, thus exposing the steel to corrosion. Also, an oral acid pH fluoride environment interferes with Ti-based devices due to the interaction of F^−^ with the titanium dioxide layer, thus providing optimal conditions for metal ions release from Ti alloys, such as V and Al. Besides this, saliva micro-organisms also play an important role in dental alloys corrosion via Redox reactions or anaerobic processes. As presented in detail by Matusiewicz H et al. [33], quantification of metal ions from different biological samples (saliva, saliva plaque, blood, serum, urine, mucosa cells, soft tissues) confirmed the release of metal ions (Cr, Fe, Ni, Ti, Co, V) from dental metal devices into the human body, however, it is still not elucidated if the number of ions released is responsible for inducing a significant harmful effect. Several studies [29,34] sustained that the concentration of ions released from dental metal devices is not high enough to induce toxic reactions in the human body, whereas other studies [35,36,37] agreed that various adverse reactions are triggered by metal ions resulting from dental devices, such as osteolysis, allergic reactions, cytotoxicity, or even mutagenic and carcinogenic processes.

Considering all the above-mentioned aspects, it is highly recommended that the various metal materials used in pediatric clinical practice be evaluated in terms of antibacterial properties, possible cytotoxic effects, and gene alterations. Regarding these aspects, the present research aimed to evaluate the effects induced by three types of space maintainers on different biological processes, such as in vitro antimicrobial behavior, cell morphology, viability, and gene expression of primary and immortalized keratinocytes associated with possible issues related to orthodontic interventions in children. Space maintainers are mainly used when premature loss of molars occurs, to prevent certain processes that can significantly alter permanent teeth. The choice of a metal medical device, such as space maintainers, depends, in particular, on the type of maintenance, the specific indications, and the age of the patient. Due to the age profile of the patients for whom these metal devices are used, there are well-founded concerns related to the development of bacterial plaque. This can be most easily controlled by optimal hygiene, however, at an early age, this is more difficult to control, and can produce much more serious effects. Human saliva contains an impressive number of bacterial cells (approximately 100 million) derived from over 700 species of bacteria and plays a key role in balancing the oral microbiome [38,39]. The variations that occur in the alteration of the oral microbiota led to the creation of an environment conducive to the development of bacteria, usually streptococci and lactobacilli, responsible for the appearance of dental issues [38]. The most commonly isolated species of Candida is *Candida albicans*, normally found in the oral flora without clinical signs of infection. In the case of orthodontic treatments, oral hygiene plays a significant role in reducing the pressure on the immune cells, which, under stress conditions, favors the development of *Candida albicans*, and leads to infections [40]. At the same time, among children, those with a low content of antimicrobial bullets in saliva are susceptible to the inception and development of candidiasis. Therefore, in this study, we tested the antimicrobial activity of salivary samples derived from the three orthodontic metal samples against positive and negative bacteria, and also fungi. S1, S2, and S3 exerted a concentration-dependent effect on *E. faecalis*, *S. mutans*, and *E. coli*, with the most active impact being observed for S2 at all three dilutions used (Figure 3). Incubation conditions related to antimicrobial assays of metallic samples used in orthodontics varies in the literature, and can be realized in aerobic, anaerobic, or CO_2_-enriched conditions at 24 and 48 h [41,42]. In the present case, there were no significant differences in incubation time, and the saliva samples tested did not stimulate the growth of microorganisms, and did not significantly inhibit their development. Other studies have shown that the use of space maintainers, both fixed and removable, can cause the growth of local microorganisms, and careful monitoring of children and proper oral hygiene are the only solutions to prevent cavities and periodontal disease [1,43].

The biocompatibility of space maintainers may be considered the most important characteristic for clinical application, especially in pediatric dentistry. In this regard, the present study evaluates the biosecurity profile of three different space maintainers by providing preliminary data on an in vitro model consisting of two different types of keratinocytes: (i) primary gingival keratinocytes (PGK); and (ii) immortalized human keratinocytes (HaCaT). The selection of the in vitro model derives from the fact that these two types of keratinocytes manifest different characteristics, mainly if referring to the re-epithelialization process initiated in the wound healing process. In the oral mucosa, wound healing develops much faster and leaves no scars, and the skin healing process is slower, aspects that could be related to higher inflammatory reactions [44] and a higher level of the most important mediator of wound angiogenesis phenomenon, the Vascular Endothelial Growth Factor (VEGF) [45,46], that is encountered in the skin wounds.

Nevertheless, the results obtained in the current study revealed that all three space maintainers induced no significant cytotoxicity on the primary gingival keratinocytes (PGK) and immortalized human keratinocytes (HaCaT) when a dilution of 1:15 (*v*:*v*%) artificial saliva:cell culture medium was applied on the cells for a period of 24 h. However, as a general remark, the primary keratinocytes were slightly more sensitive to this treatment compared to the immortalized cell line. As presented in Figure 6, the viability of the PGK cell line was reduced to 91.07% after exposure to the extraction saliva of S1, whereas the viability of HaCaT cells was 96.4%. Also, the cytotoxic rate, evaluated by means of LDH leakage method (Figure 7), presents the same pattern regarding the sensitivity of the keratinocyte cell lines to this treatment: PGK cells exhibited a higher cytotoxic rate after treatment with the extraction saliva of S1 compared to the HaCaT response after the same treatment (the cytotoxic rate being 10.84% versus 3.62%, respectively). The higher resistance expressed by the immortalized human keratinocytes (HaCaT) when compared to the primary keratinocyte cell line (PGK) may be caused by several changes developed by the immortalized cell line in order to adapt to hundreds of passages. These assumptions are in good agreement with the ones observed in a study published by Olschlager et al. [11], where the behavior of primary human fibroblasts and keratinocytes was compared to that of the immortalized cells after treatment with sodium dodecyl sulfate, revealing that primary cells are more sensitive, especially keratinocyte cultures.

However, based on the present results, no test dental metallic specimen (S1, S2, S3) should be classified as cytotoxic, as according to the ISO Standard 10993-5:2009 regarding the Biological Evaluation of Medical Devices [23], one compound is considered cytotoxic only if it reduces the viability of the treated cells by more than 30%, an effect that was not recorded for these experiments.

During the death of apoptotic cells, members of the B-cell lymphoma 2 (Bcl-2) family play a significant role, and undergo various changes. The Bcl-2 family includes a series of proteins that play crucial roles in apoptosis by regulating mitochondrial function, and are divided into two broad categories, pro-apoptotic proteins (a number of 13 in mammals including Bad, Bak, Bax, Bid, etc.); and anti-apoptotic proteins (a number 8 in mammals, including Bcl-2, Bcl-XL, Bcl-w, etc.) [47,48]. Bcl-2 expression has been identified in patients suffering from periodontal disease, but also in healthy patients, which has led to the association of inflammatory processes with the onset of periodontal disease [48]. In the case of terminal keratinocyte differentiation, Bcl-2 (anti-apoptotic marker) is subject to downward regulation upon initiation of differentiation, being expressed in undifferentiated keratinocytes. At the same time, the anti-apoptotic marker Bcl-XL is expressed in the suprabasal layers of the epidermis [49]. In this study, an increase in Bcl-XL expression in both types of keratinocytes, especially in primary gingival cells, was observed for S2, whereas the S3 sample had a very weak influence on the pro-apoptotic marker Bad, as can be seen in Figure 8. The elucidation of apoptotic mechanisms is important to quantify the influence of various physiological and pathological stimuli that intervene in both healthy and diseased bodies. Quantification of apoptosis in diseases of the oral cavity significantly helps in the selection of the optimal treatment and prevention of serious diseases. Different surface topographies of metallic materials used in pediatric dentistry have effects, especially on reducing the growth of bacterial biofilms. Therefore, future studies focusing on biofilm formation and gene expression detail are needed to contribute to the complete elucidation of the mechanisms of action and, especially, the longevity of use.

## 5. Conclusions

Space maintainers play a major role in pediatric dentistry when molars are lost prematurely and there is a risk of permanent dentition affectation. Metal devices have raised concerns due to the release of metal ions that have been shown to be possible allergens, and also shown to exert reversible toxic effects. The data of the current study bring consistent evidence that the samples evaluated are biocompatible, do not develop the growth of the studied bacteria, and do not encode the gene expression of primary and immortalized keratinocytes. However, further studies (e.g., in silico, in vivo) are required to confirm the safety of space maintainers in pediatric dentistry, especially at young age.

## Figures and Tables

**Table 1:** Microorganisms used to assess the antimicrobial effect of three saliva samples of space maintainers.

Microorganisms	Code
*Enterococcus faecalis*	ATCC^®^ 51299™
*Streptococcus mutans*	ATCC^®^ 25175™
*Escherichia coli*	ATCC^®^ 25922™
*Pseudomonas aeruginosa*	ATCC^®^ 27853™
*Candida albicans*	ATCC^®^ 10231™

**Table 2:** The oligonucleotides of the primers utilized in the experiments.

Genes	Forward Primer Sequence	Reverse Primer Sequence
18 S	5′ GTAACCCGTTGAACCCCATT 3′	5′CCA-TCC-AAT-CGG-TAGTAG-CG 3′
BCL-XL	5′GATCCCCATGGCAGCAGTAAAGCAAG3′	5′CCCCATCCCGGAAGAGTTCATTCACT 3′
Bcl-2	5′CGGGAGATGTCGCCCCTGGT 3′	5′-GCATGCTGGGGCCGTACAGT-3′
Bad	5′ CCCAGAGTTTGAGCCGAGTG 3′	5′CCCATCCCTTCGTCCT3′

## Data Availability

The data presented in this study are available on request from the corresponding author.

## References

[1] Arikan V., Kizilci E., Ozalp N., Ozcelik B. (2015). Effects of Fixed and Removable Space Maintainers on Plaque Accumulation, Periodontal Health, Candidal and Enterococcus Faecalis Carriage. Med. Princ. Pract..

[2] Setia V., Pandit I.K., Srivastava N., Gugnani N., Sekhon H.K. (2013). Space maintainers in dentistry: Past to present. J. Clin. Diagn. Res..

[3] Valm A.M. (2019). The Structure of Dental Plaque Microbial Communities in the Transition from Health to Dental Caries and Periodontal Disease. J. Mol. Biol..

[4] Saloom H.F., Mohammed-Salih H.S., Rasheed S.F. (2013). The influence of different types of fixed orthodontic appliance on the growth and adherence of microorganisms (in vitro study). J. Clin. Exp. Dent..

[5] Ireland A.J., Soro V., Sprague S.V., Harradine N.W., Day C., Al-Anezi S., Jenkinson H.F., Sherriff M., Dymock D., Sandy J.R. (2014). The effects of different orthodontic appliances upon microbial communities. Orthod. Craniofacial Res..

[6] Lucchese A., Bonini C., Noviello M., Lupo Stanghellini M.T., Greco R., Peccatori J., Biella A., Tassi E., Beretta V., Ciceri F. (2021). The Effect of Removable Orthodontic Appliances on Oral Microbiota: A Systematic Review. Appl. Sci..

[7] Alghamdi F., Shakir M. (2020). The Influence of Enterococcus faecalis as a Dental Root Canal Pathogen on Endodontic Treatment: A Systematic Review. Cureus.

[8] Xiao J., Moon Y., Li L., Rustchenko E., Wakabayashi H., Zhao X., Feng C., Gill S.R., McLaren S., Malmstrom H. (2016). Candida albicans Carriage in Children with Severe Early Childhood Caries (S-ECC) and Maternal Relatedness. PLoS ONE.

[9] Lupi S.M., Zaffe D., Rodriguez y Baena R., Rizzo S., Botticelli A.R. (2010). Cytopathological and chemico-physical analyses of smears of mucosa surrounding oral piercing. Oral Dis..

[10] Anand A., Sharma A., Kumar P., Sandhu M., Sachdeva S., Sachdev V. (2015). A Comparative Study of Biodegradation of Nickel and Chromium from Space Maintainers: An in vitro Study. Int. J. Clin. Pediatr. Dent..

[11] Satija A., Sidhu M.S., Grover S., Malik V., Yadav P., Diwakar R. (2014). Evaluation of Salivary and Serum Concentration of Nickel and Chromium Ions in Orthodontic Patients and Their Possible Influence on Hepatic Enzymes: An in vivo Study. J. Ind. Orthod. Soc..

[12] Ramakrishnan M., Dhanalakshmi R., Subramanian E. (2019). Survival rate of different fixed posterior space maintainers used in Paediatric Dentistry—A systematic review. Saudi Dent. J..

[13] Patano A., Cirulli N., Beretta M., Plantamura P., Inchingolo A.D., Inchingolo A.M., Bordea I.R., Malcangi G., Marinelli G., Scarano A. (2021). Education Technology in Orthodontics and Paediatric Dentistry during the COVID-19 Pandemic: A Systematic Review. Int. J. Environ. Res. Public Health.

[14] Bordea I.R., Sîrbu A., Lucaciu O., Ilea A., Câmpian R.S., Todea D.A., Alexescu T.G., Aluaș M., Budin C., Pop A.S. (2019). Microleakage—The Main Culprit in Bracket Bond Failure?. J. Mind Med. Sci..

[15] Kettle J.E., Hyde A.C., Frawley T., Granger C., Longstaff S.J., Benson P.E. (2020). Managing orthodontic appliances in everyday life: A qualitative study of young people’s experiences with removable functional appliances, fixed appliances and retainers. J. Orthod..

[16] Ölschläger V., Schrader A., Hockertz S. (2009). Comparison of Primary Human Fibroblasts and Keratinocytes with Immortalized Cell Lines Regarding their Sensitivity to SodiumDodecyl Sulfate in a Neutral Red Uptake Cytotoxicity Assay. Arzneimittelforschung.

[17] Fabricky M.M.C., Gabor A.G., Milutinovici R.A., Watz C.G., Avram S., Drăghici G., Mihali C.V., Moacă E.A., Dehelean C.A., Galuscan A. (2021). Scaffold-Type Structure Dental Ceramics with Different Compositions Evaluated through Physicochemical Characteristics and Biosecurity Profiles. Materials.

[18] Szuhanek C.A., Watz C.G., Avram Ș., Moacă E.-A., Mihali C.V., Popa A., Campan A.A., Nicolov M., Dehelean C.A. (2020). Comparative Toxicological In Vitro and In Ovo Screening of Different Orthodontic Implants Currently Used in Dentistry. Materials.

[19] Popa A., Dehelean C., Calniceanu H., Watz C., Brad S., Sinescu C., Marcu O.A., Popa C.S., Avram S., Nicolov M. (2020). A Custom-Made Orthodontic Mini-Implant—Effect of Insertion Angle and Cortical Bone Thickness on Stress Distribution with a Complex In Vitro and In Vivo Biosafety Profile. Materials.

[20] Dumbrava D., Popescu L.A., Soica C.M., Nicolin A., Cocan I., Negrea M., Alexa E., Obistioiu D., Radulov I., Popescu S. (2020). Nutritional, Antioxidant, Antimicrobial, and Toxicological Profile of Two Innovative Types of Vegan, Sugar-Free Chocolate. Foods.

[21] Pop D., Buzatu R., Moacă E.A., Watz C.G., Cîntă-Pînzaru S., Barbu Tudoran L., Nekvapil F., Avram Ș., Dehelean C.A., Crețu M.O. (2021). Development and Characterization of Fe3O4@Carbon Nanoparticles and Their Biological Screening Related to Oral Administration. Materials.

[22] De Andrade Lima Chaves C., de Souza Costa C.A., Vergani C.E., Chaves de Souza P.P., Machado A.L. (2014). Effects of soft denture liners on L929 fibroblasts, HaCaT keratinocytes, and RAW 264.7 macrophages. Biomed. Res. Int..

[23] ISO 10993-5:2009. Reviewed and Confirmed in 2017, Biological Evaluation of Medical Devices—Part 5: Tests for In Vitro Cytotoxicity. ISO Catalogue, Edition 3. https://www.iso.org/standard/36406.html.

[24] (2020). Performance Standards for Antimicrobial Susceptibility Testing.

[25] Popovici R.A., Vaduva D., Pinzaru I., Dehelean C.A., Farcas C.G., Coricovac D., Danciu C., Popescu I., Alexa E., Lazureanu V. (2019). A comparative study on the biological activity of essential oil and total hydro-alcoholic extract of *Satureja hortensis* L.. Exp. Ther. Med..

[26] Guran K., Buzatu R., Pinzaru I., Boruga M., Marcovici I., Coricovac D., Avram S., Poenaru M., Susan M., Susan R. (2021). In Vitro Pharmaco-Toxicological Characterization of Melissa officinalis Total Extract Using Oral, Pharynx and Colorectal Carcinoma Cell Lines. Processes.

[27] Coricovac D., Farcas C., Nica C., Pinzaru I., Simu S., Stoian D., Soica C., Proks M., Avram S., Navolan D. (2018). Ethinylestradiol and Levonorgestrel as Active Agents in Normal Skin, and Pathological Conditions Induced by UVB Exposure: In Vitro and In Ovo Assessments. Int. J. Mol. Sci..

[28] Maghiari A.L., Coricovac D., Pinzaru I.A., Macașoi I.G., Marcovici I., Simu S., Navolan D., Dehelean C. (2020). High Concentrations of Aspartame Induce Pro-Angiogenic Effects in Ovo and Cytotoxic Effects in HT-29 Human Colorectal Carcinoma Cells. Nutrients.

[29] Chen Z., Patwari M., Liu D. (2019). Cytotoxicity of orthodontic temporary anchorage devices on hu-man periodontal ligament fibroblasts in vitro. Clin. Exp. Dent. Res..

[30] Mikulewicz M., Chojnacka K., Wołowiec P. (2014). Release of metal ions from fixed orthodontic appliance: An in vitro study in continuous flow system. Angle Orthod..

[31] Hanawa T., Ni-inomi M. (2019). Overview of metals and applications. Biomaterials, Metals for Biomedical Devices.

[32] Fernández-Miñano E., Ortiz C., Vicente A., Calvo Guirado J.L., Ortiz A.J. (2011). Metallic ion content and damage to the DNA in oral mucosa cells of children with fixed orthodontic appliances. Biometals.

[33] Matusiewicz H. (2014). Potential release of in vivo trace metals from metallic medical im-plants in the human body: From ions to nanoparticles--a systematic analytical review. Acta Biomater..

[34] Martin-Camean A., Jos A., Puerto M., Calleja A., Iglesias-Linares A., Solano E., Camean A.M. (2015). In vivo determination of Aluminum, Cobalt, Chromium, Copper, Nick-el, Titanium and Vanadium in oral mucosa cells from orthodontic patients with mini-implants by Inductively coupled plasma-mass spectrometry (ICP-MS). J. Trace Elem. Med. Biol..

[35] Malkoc S., Ozturk F., Corekci B., Bozkurt B.S., Hakki S.S. (2012). Real-time cell analysis of the cytotoxicity of orthodontic mini-implants on human gingival fibroblasts and mouse osteoblasts. Am. J. Orthod. Dentofacial. Orthop..

[36] Pillai A.R., Gangadharan A., Gangadharan J., Kumar N.V. (2013). Cytotoxic effects of the nickel release from the stainless steel brackets: An in vitro study. J. Pharm. Bioall. Sci..

[37] Hafez H.S., Selim E.M.N., Eid F.H.K., Tawfik W.A., Al-Ashkar E.A., Mostafa Y.A. (2011). Cytotoxicity, genotoxicity, and metal release in patients with fixed orthodontic appli-ances: A longitudinal in-vivo study. Am. J. Orthod. Dentofac. Orthop..

[38] Kilian M., Chapple I.L., Hannig M., Marsh P.D., Meuric V., Pedersen A.M.L., Tonetti M.S., Wade W.G., Zaura E. (2016). The oral microbiome—An update for oral healthcare professionals. Br. Dent. J..

[39] Vila T., Rizk A.M., Sultan A.S., Jabra-Rizk M.A. (2019). The power of saliva: Antimicrobial and beyond. PLoS Pathog..

[40] Farronato G., Giannini L., Galbiati G., Cannalire P., Martinelli G., Tubertini I., Maspero C. (2013). Oral tissues and orthodontic treatment: Common side effects. Minerva Stomatol..

[41] Imazato S., Kuramoto A., Takahashi Y., Ebisu S., Peters M.C. (2006). In vitro antibacterial effects of the dentin primer of Clearfil Protect Bond. Dent Mater..

[42] Brambilla E., Cagetti M.G., Gagliani M., Fadini L., García-Godoy F., Strohmenger L. (2005). Influence of different adhesive restorative materials on mutans streptococci colonization. Am. J. Dent..

[43] Gurcan A.T., Koruyucu M., Kuru S., Sepet E., Seymen F. (2021). Effects of Fixed and Removable Space Maintainers on DentalPlaque and DMFT/dft Values. Int. J. Dent. Sci..

[44] Turabelidze A., Guo S., Chung A.Y., Chen L., Dai Y., Marucha P.T., DiPietro L.A. (2014). Intrinsic differences between oral and skin keratinocytes. PLoS ONE.

[45] Chen L., Gajendrareddy P.K., DiPietro L.A. (2012). Differential expression of HIF-1α in skin and mucosal wounds. J. Dent. Res..

[46] Szpaderska A.M., Walsh C.G., Steinberg M.J., DiPietro L.A. (2005). Distinct Patterns of Angiogenesis in Oral and Skin Wounds. J. Dent. Res..

[47] Misra A., Rai S., Misra D. (2016). Functional role of apoptosis in oral diseases: An update. J. Oral Maxillofac. Pathol..

[48] Jain M., Kasetty S., Sridhara S.U., Jain N., Khan S., Desai A. (2013). Apoptosis and Its Significance in Oral Diseases: An Update. J. Oral Dis..

[49] Lippens S., Denecker G., Ovaere P., Vandenabeele P., Declercq W. (2005). Death penalty for keratinocytes: Apoptosis versus cornification. Cell Death Differ..

